# Immunomodulatory Activity of Red Ginseng against Influenza A Virus Infection

**DOI:** 10.3390/nu6020517

**Published:** 2014-01-27

**Authors:** Jong Seok Lee, Hye Suk Hwang, Eun-Ju Ko, Yu-Na Lee, Young-Man Kwon, Min-Chul Kim, Sang-Moo Kang

**Affiliations:** 1 Center for Inflammatory, Immunity & Infection, Institute for Biomedical Sciences, Georgia State University, Atlanta, GA 30303, USA; E-Mails: jongseoklee78@gmail.com (J.S.L.); hshwang33@gmail.com (H.S.H.); ej.ko226@gmail.com (E.-J.K.); mistybluerain7@gmail.com (Y.-N.L.); ymankwon@gmail.com (Y.-M.K.); mckim001@gmail.com (M.-C.K.); 2 Animal and Plant Quarantine Agency, Anyang City, Gyeonggi-do 430-757, Korea

**Keywords:** *Panax ginseng*, influenza A virus, epithelial cells, oxidative stress, interferon

## Abstract

Ginseng herbal medicine has been known to have beneficial effects on improving human health. We investigated whether red ginseng extract (RGE) has preventive effects on influenza A virus infection *in vivo* and *in vitro*. RGE was found to improve survival of human lung epithelial cells upon influenza virus infection. Also, RGE treatment reduced the expression of pro-inflammatory genes (IL-6, IL-8) probably in part through interference with the formation of reactive oxygen species by influenza A virus infection. Long-term oral administration of mice with RGE showed multiple immunomodulatory effects such as stimulating antiviral cytokine IFN-γ production after influenza A virus infection. In addition, RGE administration in mice inhibited the infiltration of inflammatory cells into the bronchial lumens. Therefore, RGE might have the potential beneficial effects on preventing influenza A virus infections via its multiple immunomodulatory functions.

## 1. Introduction

Influenza A virus is an important respiratory tract pathogen that causes seasonal epidemics and pandemics of significant morbidity and mortality. Diverse influenza A viruses with different combination of hemagglutinin (HA; 16 subtypes) and neuraminidase (NA; 9 subtypes) have been identified [1]. Vaccination is known to be an effective method preventing influenza disease. However, current vaccines are not highly protective if antigenically different new strains emerge such as the recent outbreak of the 2009 pandemic H1N1 virus [2,3]. Therefore, it is highly desirable to find a preventive measure that would have protective activity against the emergence of an unexpected influenza strain. Although the mechanisms by which influenza virus infection causes severe morbidity and mortality remain unclear, it is likely that multiple mechanisms involve in the process of influenza disease. It has been suggested that production of reactive oxygen species (ROS) caused by influenza A virus infection contributes to inducing abnormal signal transduction pathways stimulating the production of cytokines/chemokines, epithelial cell death, airway inflammation, and pulmonary damage [4,5,6,7]. These studies suggested that hypercytokinemia and epithelial cell apoptosis might play a central role in the pathogenesis of influenza-induced epithelial dysfunction.

Medicinal herbs can provide a source for finding novel anti-inflammatory agents [8,9]. *Panax ginseng* is one of the most commonly used herbal medicines in humans. *Panax ginseng* was also reported to enhance the host immune system by stimulating natural killer cells, T-cells, B-cells, and dendritic cells-dependent immune system response [10]. In addition, whole ginseng extracts and ginseng components were demonstrated to inhibit ROS-induced oxidative stress and to modulate the antioxidant defense systems [11,12]. In our previous study, administration of mice with ginseng extracts prior to infection was found to confer a survival benefit against infection with influenza A viruses [13,14]. However, the potential roles of ginseng extracts in conferring protection against influenza A virus infection *in vitro* and *in vivo* largely remain unknown.

In this study, we investigated the effect of red ginseng extract (RGE) on cell survival, cytokine expression, and cellular oxidative stress upon infection of human epithelial cells with influenza virus. In addition, we evaluated potential immunomodulatory functions of RGE oral administration upon influenza A virus infection in a mouse model.

## 2. Materials and Methods

### 2.1. Cells, Virus, Reagents

The influenza subtype H1N1 A/PR/8/34 (A/PR8) and A/WSN/1933 viruses were described in previous studies [13,14]. Influenza viruses were grown in 11-day old embryonated hen’s eggs. Egg allantonic fluids were harvested and stored at −80 °C until use. A549 cells, a human alveolar type II-like epithelial cell line, were generous gifts from Dr Jae Hyang Lim (Center for Inflammation, Immunity & Infection, Georgia State University). Mice were infected with serial dilutions of influenza virus and the 50% lethal dose (LD_50_) was determined. Korean red ginseng extract (RGE), a concentrated form of the commercial ginseng product was kindly provided by Korea Ginseng Corporation (Daejeon, Korea). Briefly, fresh roots of the *Panax ginseng* that had grown for 6 years were washed, steamed at 100 °C for 2 to 3 h and dried. The dried red ginseng roots were boiled in 4 to 5 volumes of water for 3 h and the supernatants were concentrated. This preparation was designated “RGE” (approximately 36% water content). Fetal bovine serum (FBS), penicillin–streptomycin, and F12K Nutrient Mixture were purchased from GIBCO (Grand Island, NY, USA). Dichlorodihydrofluorescein diacetate (H_2_DCFDA) was purchased from Molecular Probes (Carlsbad, CA, USA). All other chemicals were analytical grade.

### 2.2. Cell Viability and Cytopathogenic Effect (CPE) Reduction Assays

The effect of RGE and H1N1 influenza A virus on the viability of the cells was measured using the [3-(4,5-dimethylthiazol-2-yl)-2,5-diphenyltetrazolium] bromide (MTT) assay, which relies on the ability of viable cells to metabolically reduce the tetrazolium salt MTT to a purple formazan product, which can be quantified colorimetrically [15]. In brief, A549 cells were continuously treated with RGE starting with 24 h pre-incubation period. After virus infection, cells were treated with or without RGE for another 48 h. After various treatments, 50 μL of the MTT stock solution (2 mg/mL) was then added to each well to attain a total reaction volume of 200 μL. After incubation for 2 h at 37 °C, the formazan crystals in each well were dissolved in isopropyl alcohol, and the absorbance was determined at 570 nm. The relative percentage of cell viability was calculated with the control cells treated with vehicle as 100%. The cytopathogenic effect (CPE) reduction assay was determined as previously described [16]. Confluent cell monolayers were infected with influenza A virus at the indicated multiplicities of infection (MOIs). After 1 h adsorption period, cells were washed to remove non-detached virus. The virus-induced CPE was recorded at 48 h post infection. A549 cells were continuously treated with RGE starting 1 day prior to infection and during the infection period.

### 2.3. Reverse Transcriptase-Polymerase Chain Reaction (RT-PCR)

Total RNA was isolated from cell pellets using the RNeasy mini kit system (QIAGEN, Valencia, CA, USA) according to the manufacturer’s instruction, and treated with DNase I to remove traces of contaminant DNA. The relative amount of each interest gene was determined by semiquantitative RT-PCR, as previously described [17]. Briefly, first strand cDNA synthesis utilized total RNA (1 μg) from each sample, oligo dT primers, and Super Script III RT (Invitrogen, Carlsbad, CA, USA). mRNA level of IL-6, IL-8 and 18S rRNA was determined by using following oligonucleotides as primers; IL-6 sense 5′-GACAGCCACTCACCTCTTCA-3′, anti-sense 5′-CATCTTTGGAAGGTTCAGGTTGT-3′, IL-8 sense 5′-CAGCCTTCCTGATTTCTGC-3′, anti-sense 5′-ACTTCTCCACAACCCTCTGC-3′, 18S rRNA sense 5′-ATCCTGCCAGTAGCATATGC-3′, anti-sense 5′-ACCCGGGTTGGTTTTGATCTG-3′. RT-PCR products were analyzed on 2% agarose gels, and bands were visualized by ethidium bromide staining. As an internal control for equal mRNA expression, 18S rRNA was used. The relative amount of RT-PCR products was expressed as fold increase relative to mock controls.

### 2.4. Intracellular ROS Measurement and Image Analysis

To investigate the effect of RGE on oxidative stress, the fluorescent probe H_2_DCFDA was used to evaluate the level of intracellular ROS [18]. Briefly, after various treatments, cells were incubated with 5 μM H_2_DCFDA solution in PBS for 2 h at 37 °C. The degree of fluorescence was measured at an excitation and emission wavelength of 485 and 535 nm, respectively, by a microplate spectrofluorometer. For image analysis of the production of intracellular ROS, cells were seeded in coverslip-loaded six well plates. After various treatments, cells were fixed with 3.7% paraformaldehyde for 20 min and washed with PBS. H_2_DCFDA solution was added to each well of the plate, which was incubated for 2 h at 37 °C. Images of the stained cells were collected using a fluorescence microscope (Nikon, Tokyo, Japan).

### 2.5. Oral Administration of Mice with RGE and Influenza A Virus Infection

RGE was dissolved in sterile PBS and filtered through 0.4 μm Millipore membrane. For animal experiments, 9–10 months old female BALB/c mice (Harlan Laboratories, Indianapolis, IN, USA) were lightly anesthetized by isoflurane and then RGE was administered orally at a dose of 25 mg/kg/day for 30 days. Oral administration was carried out using a 0.9 × 39 mm stainless steel feeding needle with a silicone tip to avoid damage to the esophagus and trachea. To determine the effects of RGE treatment on H1N1 influenza A virus infection, mice (*n* = 5 per group) were anesthetized by isoflurane inhalation and intranasally infected with a mouse-adapted pathogenic influenza A H1N1 virus A/PR/8/34 (1.0 LD_50_). Mice were monitored daily to record weight changes. All animal experiments presented in this manuscript were approved by the Georgia State University (GSU) Institutional Animal Care and Use Committee review board.

### 2.6. Cytokine Assays

The individual lungs were removed aseptically at day 5 post challenge, and lung extract were prepared as homogenates after challenge using frosted glass slides. The homogenates were centrifuged at 2000 rpm for 10 min to collect supernatants. The lung supernatants were frozen and kept at −80 °C until used for cytokine assays. Cytokine ELISA was performed as described previously [19]. Ready-Set-Go IL-6 and IFN-γ kits (eBioscience, San Diego, CA, USA) were used for detecting cytokine levels in lung extracts and bronchoalveolar (BAL) fluids following the manufacturer’s recommended procedures.

### 2.7. Pulmonary Histology of Influenza Virus-Infected Mice

For histological analysis of lung tissues, mice were anesthetized with isoflurane and exsanguinated after severing of the right caudal artery. The lungs were then fixed via infusion through the trachea with 4% formalin, removed, immersed in 4% formalin for 24 h, embedded in paraffin, sectioned, and stained with hematoxylin and eosin (H & E) or periodic acid-Schiff stain (PAS). Ten sections per mouse were obtained. For sections stained with H & E, the percentage of intrabronchiolar exudate positive area in 25 randomly selected airways was determined by Adobe Photoshop CS5.1 software [20].

### 2.8. Preparation of Bronchoalveolar Lavage (BAL) and Flow Cytometric Analysis

Five days after RSV infection, mice were sacrificed to collect BAL fluids and lung samples. BAL fluid samples were obtained by infusing 1 mL of PBS into the lungs via the trachea using a 25-gauge catheter (Exelint International Co., Los Angeles, CA, USA). Cells from BAL fluids were pooled (*n* = 5) and then stimulated with phorbol myristate acetate (50 ng/mL) and ionomycin (500 ng/mL) for 4 h. After staining with surface antibodies (anti-CD45, CD3, CD4, CD8α, CD11b, and CD11c antibodies from eBiosciences), intracellular IFN-γ cytokine staining was followed by manufacturer’s manuals (BD Cytofix/Cytoperm™ Fixation/Permeabilization Solution Kit, San Diego, CA, USA). The percentage of gated cells was calculated by Flow Jo software (Tree Star Inc., Ashland, OR, USA) [21].

### 2.9. Statistical Analysis

Data were expressed as means ± standard error (SEM), and the results were taken from at least three independent experiments performed in triplicate. The data were analyzed by Student’s *t*-test to evaluate significant differences. Nonparametric ANOVA tests were also performed for comparing multiple groups when appropriate. A level of *p* < 0.05 was regarded as statistically significant.

## 3. Results

### 3.1. RGE Improves Viability of Human Epithelial Cells upon Infection with Influenza A Virus

**Figure 1:**
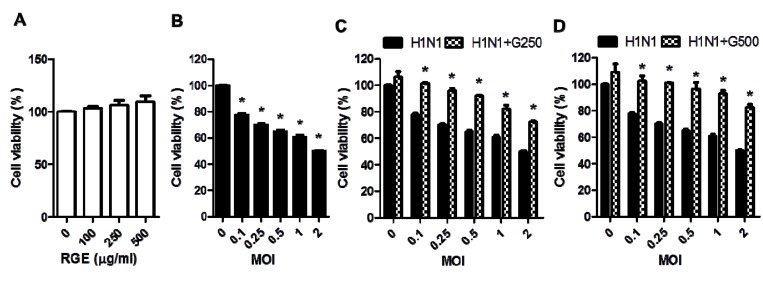
Influence of red ginseng extract (RGE) on H1N1 influenza A virus-induced cytopathogenic effect (CPE) formation in A549 cells. (**A**) Influence of different RGE concentrations on the growth of A549 cells. Values are the mean ± SEM. * *p* < 0.05 *vs*. mock control; (**B**) Influence of different H1N1 influenza A virus MOIs on the growth of A549 cells. Values are the mean ± SEM. * *p* < 0.05 *vs*. mock control; (**C**,**D**) Influence of different RGE concentrations on the growth of A549 cells infected with H1N1 influenza A virus at different MOIs 48 h post infection. A549 cells were continuously treated with RGE starting with 24 h pre-incubation period. Cell viability was measured by MTT assay. Values are the mean ± SEM. * *p* < 0.05 *vs*. H1N1 influenza A virus-infected group.

**Figure 2:**
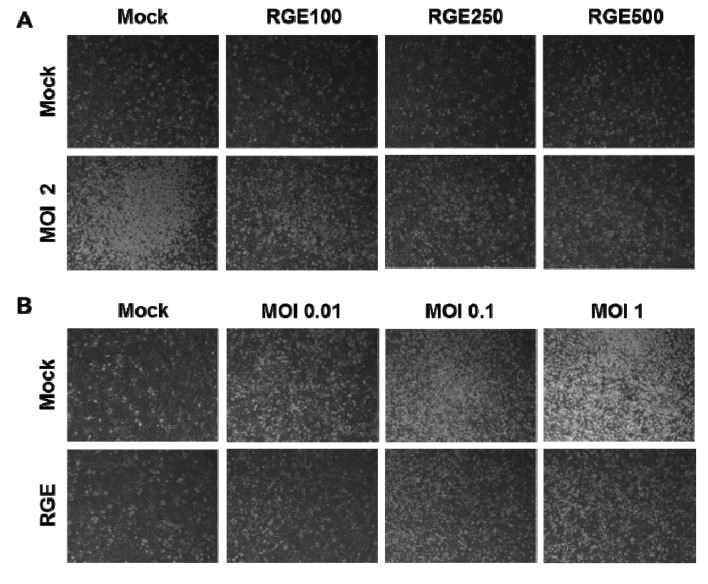
Representative pictures of CPE formation in A549 cells infected with H1N1 influenza A virus at different multiplicities of infection (MOIs) without or with RGE. A549 cells were mock-infected or infected with H1N1 influenza A virus at different MOIs 48 h post infection. A549 cells were continuously treated with RGE starting with 24 h pre-incubation period. (**A**) Influence of different RGE concentrations on A549 cells infected with H1N1 influenza A virus at a MOI of 2; (**B**) Influence of RGE (500 μg/mL) on A549 cells infected with H1N1 influenza A virus at different MOI from 0 to 1.

RGE itself did not affect the viability of human epithelial cells (Figure 1A). To investigate whether red ginseng extract (RGE) would protect epithelial cells against influenza A viral infection, human alveolar epithelial A549 cells were infected with H1N1 influenza A virus (A/WSN/33) at different MOIs in the presence or absence RGE treatment. The influenza H1N1 virus A/WSN/33 was found to induce severe cytopathogenic effects in a dose-dependent manner (Figure 1 and Figure 2). Upon infection of A549 cells with influenza A virus, human epithelial cells showed pronounced morphology of cytopathogenic formation, such as cell rounding and cell detachment leading to cell death (Figure 2). This is consistent with results of previous studies demonstrating the apoptosis of cells infected with influenza A virus [16,22]. Interestingly, human epithelial A549 cells treated with RGE remarkably decreased viral cytopathogenic effects and reduced cell death caused by influenza A H1N1 virus infection (Figure 1 and Figure 2). These protective effects by RGE were more pronounced at higher MOIs (Figure 1C,D). Therefore, these results suggest that RGE improves viability of human epithelial cells upon H1N1 influenza A virus infection.

### 3.2. RGE Treatment Reduces Influenza A Virus-Induced Cytokine Production

**Figure 3:**
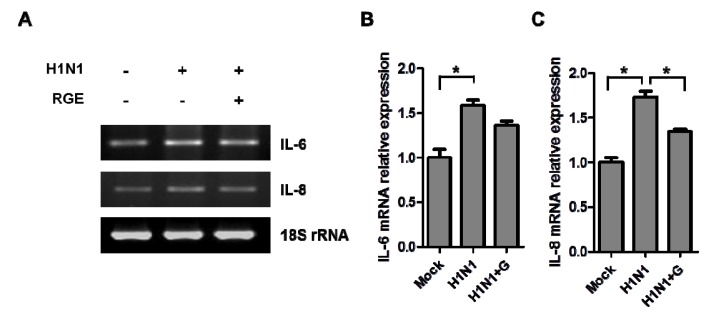
Influence of RGE on H1N1 influenza A virus-induced cytokine production in A549 cells. (**A**) Representative RT-PCR bands of cytokine mRNA expression. (**B** and **C**) The relative quantity of cytokine mRNA expression. A549 cells were mock-infected or infected with H1N1 influenza A virus. A549 cells were continuously treated with RGE (250 μg/mL) starting with 24 h pre-incubation period. After virus infection, cells were treated with or without RGE for another 48 h. Cytokine mRNA levels were determined by semiquantitative RT-PCR. Values are the mean ± SEM. * *p* < 0.05.

Influenza infections cause airway epithelial inflammation and oxidant-mediated cell damage [23]. To investigate the effects of RGE on inflammatory responses of epithelial cells to influenza A virus infection, we determined the expression of cytokine IL-6 and chemokine IL-8. Influenza A virus at a MOI of 1 in the presence or absence RGE treatment was inoculated onto confluent human epithelial A549 cell layers. Total RNA of human epithelial cells was extracted and the gene expression was evaluated by RT-PCR. Human epithelial cells infected with influenza A virus significantly increased the expression of IL-6 and IL-8 compared to mock-treated cells and RGE treatment inhibited IL-6 and IL-8 production induced by influenza A virus-infected human epithelial cells (Figure 3). These results indicate that RGE may suppress inflammatory responses in lung alveolar epithelial cells upon infection with influenza A virus.

### 3.3. RGE Inhibits Influenza A Virus-Induced ROS Formation

**Figure 4:**
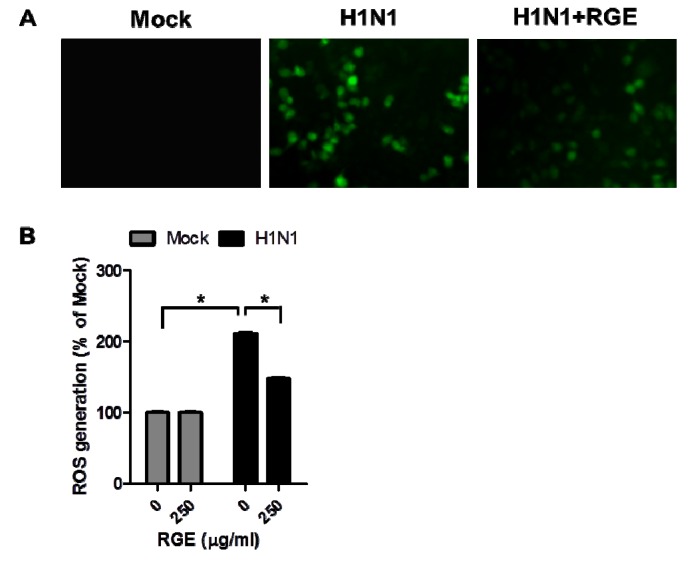
Influence of RGE on H1N1 influenza A virus-induced ROS formation in A549 cells. A549 cells were mock-infected or infected with H1N1 influenza A virus. A549 cells were continuously treated with RGE (250 μg/mL) starting with 24 h pre-incubation period. After virus infection, cells were treated with or without RGE for another 48 h. (**A**) Fluorescence microscopic image of H1N1 influenza A virus-induced ROS formation. ROS level of cells was detected by the fluorescence microscope; (**B**) The extent of H1N1 influenza A virus-induced ROS generation. The degree of fluorescence was detected at 485 nm excitation and at 535 nm emission using a microplate spectrofluorometer. Values are the mean ± SEM. * *p* < 0.05.

Infection of cells with influenza A virus was reported to diminish cellular glutathione [23]. Oxidative stress mediated-events are known to be involved in the virus infection mechanisms and correlated with release of inflammatory mediators from infected human epithelial cells [16,24]. To investigate the potential effects of RGE on the oxidative stress mediated-events in response to influenza A virus infection, we determined the levels of ROS in infected A549 cells. Influenza A virus at a MOI of 1 in the presence or absence RGE was inoculated onto confluent human epithelial cell layers. The ROS-sensitive fluorescent probe H_2_DCFDA was used to determine intracellular ROS levels in influenza A virus-infected human epithelial cells. RGE did not affect ROS generation of un-infected A549 cells. The fluorescent intensity of the DCFDA stain was enhanced in influenza A virus-infected human epithelial A549 cells, indicating a significant amount of ROS generation due to influenza A virus infection. However, RGE significantly diminished the generation of ROS as evidenced by a low level of the green fluorescence intensity upon influenza A virus infection (Figure 4). These results suggest that RGE may interfere with oxidative stress events associated with influenza A virus infection.

### 3.4. Oral Administration of Mice with RGE Contributes to Reducing Pulmonary Inflammation upon Influenza A Virus Infection

Most ginseng products are consumed orally in humans. We have shown that oral administration of mice with RGE conferred survival protective benefits upon influenza A virus infection [13,14]. To better understand RGE-mediated protective benefits on influenza A virus infection, we tested the effects of RGE on virus-induced inflammation in a mouse model. BALB/c mice were orally administered RGE at a dose of 25 mg/kg/day for 30 days.

**Figure 5:**
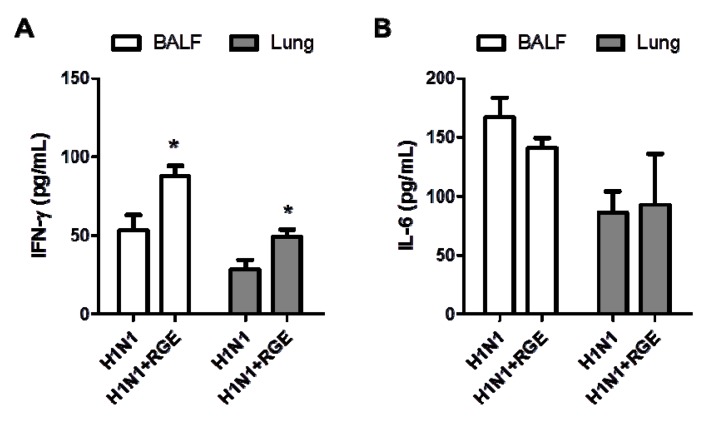
Influence of RGE on cytokines in mice infected with H1N1 influenza A virus. Cytokine IFN-γ (**A**) and IL-6 (**B**) levels of lung extract and bronchoalveolar lavage fluids (BALF) were determined at day 5 post infection (*n* = 5). H1N1: naïve mice challenged with H1N1 influenza virus, H1N1 + RGE: RGE was administered orally at a dose of 25 mg/kg/day for 30 days and then mice were challenged with H1N1 influenza A virus. Values are the mean ± SEM. * *p* < 0.05 *vs*. H1N1-infected group.

**Figure 6:**
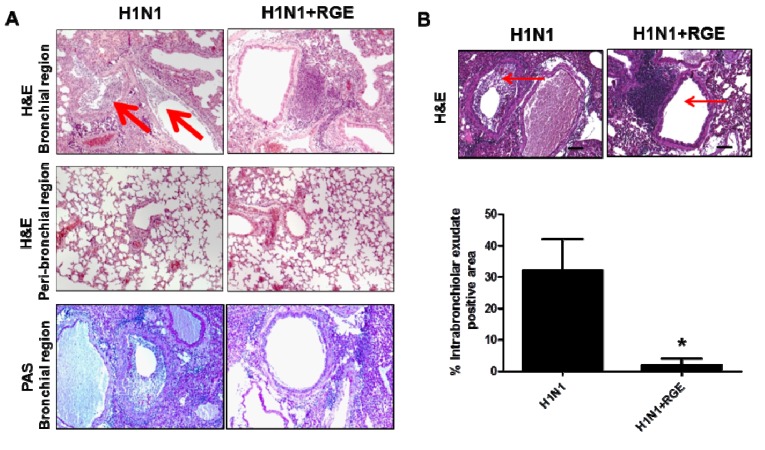
Influence of RGE on pulmonary histopathology in mice infected with H1N1 influenza A virus. (**A**) HE and PAS staining of lung tissues. (**B**) Intrabronchiolar exudates of necrotic cell debris. RGE was administered orally at a dose of 25 mg/kg/day for 30 days and then mice were challenged with H1N1 influenza A virus. Lungs were prepared for tissue sections, as described in Materials and Methods, and stained with H & E or PAS. All images were acquired using the same camera settings. Images were acquired using a Zeiss Axiovert 100 microscope at a magnification of 100×, using an attached Canon 30D digital camera.

RGE-treated or untreated mice were intranasally infected with a mouse-adapted pathogenic influenza A H1N1 virus A/PR/8/34 (1.0 LD_50_). Lungs and BAL fluid samples were collected at day 5 post infection to determine cytokine levels. The level of cytokine IL-6 in lungs and BAL samples from RGE treated mice was moderately lower than or similar to that from untreated mice following influenza A virus infection. In contrast, IFN-γ was detected at significantly higher levels in both lungs and BAL samples from RGE-administered mice compared to those from untreated control mice upon influenza A virus infection (Figure 5). In support of this high IFN-γ cytokine levels by RGE treatment, we observed that RGE treatment moderately increased the cellularity of CD8 T cells and CD11c^+^ phenotype cells producing IFN-γ in BAL samples (data not shown). Lung histopathology was also assessed following intranasal influenza A virus infection of RGE oral-treated mice. Histological H & E and PAS stained slides were prepared from lung tissue 5 days post infection. PAS staining did not show a significant difference in both infected naïve mice with or without RGE. We observed infiltration of abundant inflammatory cells into the bronchial lumens and luminal cell infiltration of influenza A virus infected naïve control mice (Figure 6A). Influenza virus infection resulted in severe inflammation of bronchioles which are narrowest airways of the lung. The bronchioles appeared to be flooded and surrounded with polymorphonuclear inflammatory cells infiltrating into airways. The degrees of inflammation of bronchioles were determined by percentages of bronchiolar exudate positive area in whole bronchiole area with randomly selected airways. The RGE treatment prior to influenza virus infection significantly decreased the inflammatory exudate area in bronchioles compared to the group of influenza virus infection only without RGE (Figure 6B).

## 4. Discussion

Immunomodulation is regarded as one of the important strategies to improve the body’s defense mechanism against viral infection. There is a significant amount of experimental evidence suggesting ginseng has various immunomodulatory functions [10,11,12]. However, the potential roles of ginseng in conferring protection against inflammatory viral infection have not been well demonstrated. Here, we showed that RGE inhibited influenza A virus-induced cell death, expression of pro-inflammatory cytokines, and ROS formation in human epithelial cells. Oral administration of RGE to mice conferred immunomodulatory effects such as the production of IFN-γ upon infection with influenza A virus. Therefore, intake of ginseng might have a role in conferring protective effects on influenza A virus infection by increasing cell survival, and reducing the production of inflammatory cytokines and ROS formation, resulting in less inflammatory pulmonary disease.

It has been suggested that the severity of human respiratory disease by viral infection has been associated with pro-inflammatory hypercytokinemia [24]. Influenza A viruses were shown to induce expression of cytokines and chemokines, including CXCL8 (also known as IL-8), IL-6, CXCL10 (also known as interferon-inducible cytokine IP-10), and CCL5 (also known as RANTES) in airway epithelial cells [25,26]. *Panax ginseng* appears to have multiple biological and immunomodulatory effects. Our previous studies demonstrated that short-term daily oral treatment of mice with ginseng extract showed enhanced survival rates and lower levels of lung viral titers after infection with the 2009 pandemic H1N1 virus. Administration of mice with ginseng extract prior to infection was found to confer a survival benefit against infection with H1N1 (A/PR/8/34) and H3N2 (A/Philippines/82) influenza viruses. In addition, inactivated influenza A virus-immunized mice that were co-administered ginseng showed significant enhancement of influenza virus specific IgA and IgG antibodies in lung after challenge virus infection suggesting a role as mucosal adjuvants against influenza virus [13,14,19]. In this study, our data showed that long-term oral administration with RGE prior to infection exhibited immunomodulatory functions during H1N1 influenza A infection in naïve mice. Pre-treatment with RGE prior to infection contributed to protective immunity by stimulating T cells and CD11c^+^ phenotype cells resulting in enhanced production of IFN-γ after influenza A viral infection in a mouse model. RGE treatment appeared to diminish infiltration of inflammatory cells into the bronchial lumens and lumen cells infiltration upon influenza A virus-infection. Therefore, this study suggests that anti-inflammatory effects on reducing IL-6 and IL-8 cytokine production exerted by ginseng may be beneficial for the prevention of severe disease by influenza A virus infection, contributing to survival benefits at a moderate level.

Inflammatory responses may be responsible for the pathological signs in humans infected with influenza viruses [27]. Oxidative stress plays an important role in the pathogenesis of lung inflammation. Different sources of ROS have been suggested in influenza virus-infected lungs, and lung epithelial cells are likely to be a source of ROS production probably due to influenza virus infection-induced oxidative stress response [5]. Influenza virus-induced cellular oxidative damage is the result of an imbalance between ROS production and antioxidant cellular defenses. Whereas influenza viral infection induced a significant increase of manganese superoxide dismutase (MnSOD), influenza viral infection decreased catalase gene expression [23]. Some evidence provided that ROS as important regulators of influenza A virus-induced cellular signaling leading to the expression of key pro-inflammatory mediators, such as cytokines and chemokines. Cytokines and chemokines involved in host response to influenza infection are thought to play a central role in the pathogenesis [28]. These effects have been associated with the activation of oxidant sensitive pathways such as mitogen-activated protein kinase (MAPK) and the transcription factor nuclear factor-κB (NF-κB) [29]. In this study, our data indicated that levels of intracellular ROS production as well as IL-8 and IL-6 inflammatory cytokines were significantly increased as a result of influenza A virus infection. We found that RGE reduced influenza A virus-induced CPE formation and blocked the induction of influenza A virus-induced pro-inflammatory gene expression. Although the mechanism is not clear, RGE might at least in part interfere with influenza A virus-induced cellular oxidative damage in human alveolar type II-like epithelial cell line. Our previous data indicated that ginseng had the anti-viral growth activity against influenza A virus *in vitro* and *in vivo* [13,14]. This antioxidant effect on the virus replication has been described for other antioxidants like *N*-acetyl-l-cysteine (NAC), oligonol, and glycyrrhizin [30,31,32].

## 5. Conclusions

In conclusion, modulation of oxidative stress represents a potential novel pharmacologic approach to ameliorate influenza A virus-induced acute lung inflammation. RGE treatment inhibited influenza A virus-induced cellular oxidative damage and blocked the induction of influenza A virus-induced pro-inflammatory gene expression in human alveolar type II-like epithelial cell line. Furthermore, RGE contributed to protective immunity by enhancing the production of IFN-γ and partially blocking severe infiltration of abundant inflammatory cells upon influenza A viral infection in an experimental mouse model. Based on these results, although the exact underlying anti-virus mechanism of ginseng is yet unknown, ginseng might be able to affect influenza A virus disease by antioxidative and immunomodulatory effects.
